# Emotion dysregulation and depressive symptoms mediate the association between inhibitory control difficulties and aggressive behaviour in children with ADHD

**DOI:** 10.3389/fpsyt.2024.1329401

**Published:** 2024-04-16

**Authors:** Sofia Marques, Teresa Correia-de-Sá, Micaela Guardiano, Benedita Sampaio-Maia, Joana Ferreira-Gomes

**Affiliations:** ^1^ Institute of Psychology and Educational Sciences, Lusíada University, Porto, Portugal; ^2^ CIPD—Psychology for Development Research Centre, Lusíada University, Porto, Portugal; ^3^ Department of Biomedicine, Faculty of Medicine, University of Porto, Porto, Portugal; ^4^ INEB—Institute of Biomedical Engineering, University of Porto, Porto, Portugal; ^5^ i3S—Institute for Research & Innovation in Health, University of Porto, Porto, Portugal; ^6^ Department of Paediatrics, Unit of Neurodevelopmental Paediatrics, University Hospital Centre of São João, Porto, Portugal; ^7^ Faculty of Dental Medicine, University of Porto, Porto, Portugal; ^8^ IBMC—Institute for Molecular and Cell Biology, University of Porto, Porto, Portugal

**Keywords:** attention-deficit/hyperactivity disorder (ADHD), emotional lability, emotion dysregulation, depressive symptoms, externalising problems, inhibitory control, aggressive behaviour, mediation models

## Abstract

**Background/objectives:**

Impulsive aggressive behaviour, although not a core symptom, is often part of the clinical presentation of attention-deficit/hyperactivity disorder (ADHD). Recently, impulsive aggression has been attributed to emotion dysregulation, which is currently conceptualised as a transdiagnostic factor and seems to contribute to the co-occurrence of other problems in ADHD. Thus, this study investigated the presence of impulsive aggressive behaviour and explored whether emotion dysregulation mediates the relationship between inhibitory control difficulties and aggressive behaviour in children with ADHD. Because ADHD may act as a risk factor for the development of other conditions, such as internalising problems, we aimed to understand whether depressive symptoms contribute to this relationship.

**Methods:**

Seventy-two children were recruited from a hospital and the community, 38 of whom had ADHD and 34 were typically developing (TD). Parents completed the Child Behaviour Checklist, the Behaviour Rating Inventory of Executive Function, and the Emotion Regulation Checklist. Simple mediation and serial mediation models were performed to test our hypotheses.

**Results:**

Aggressive behaviour was significantly higher in ADHD children compared to TD children. Emotion dysregulation fully mediated the relationship between inhibitory control difficulties and aggressive behaviour in ADHD children. Adding depressive symptoms to the model increased the explained variance in aggressive behaviour.

**Conclusion:**

The main result of our study supports the role of emotion dysregulation and depressive symptoms in mediating the relationship between inhibitory control difficulties and impulsive aggressive behaviour in children with ADHD. This highlights that aggressive behaviour is, in part, a result of the inability of the child to appropriately regulate their emotions. Future interventions may be tailored to improve emotion regulation skills to address aggressive behaviour.

## Introduction

Attention-deficit/hyperactivity disorder (ADHD) is the most common neurodevelopmental disorder, affecting 5.9% of youth and 2.8% of adults (1), since in many cases symptoms persist into adulthood (2). It is characterised by varying combinations of inattention, hyperactivity, and/or impulsivity that are age-inappropriate, persistent, and pervasive, with marked impairments in academic and family functioning and peer and social relationships (3, 4). Externalising problems, such as aggressive behaviour, and ADHD are highly co-occurring (5–7). Although aggression is considered normative when children need to express anger, its use tends to decline as the child’s cognitive, language and emotion regulation abilities increase throughout the course of development (8). On the other hand, maladaptive aggressive behaviours refer to a range of behaviours that involve acting out, hostility, and aggression toward others or objects, which is persistent and disproportionate to the preceding stimulus (7). Aggression can be divided into two different categories, depending on what motivated the behaviour: i) reactive or impulsive and ii) proactive or instrumental (9). Impulsive aggression is an unplanned and spontaneous response to a true or perceived insult, reflecting lack of emotional control, and emerging out of frustration, exasperation, or hostility (7, 9). Although aggression is not a core symptom of ADHD, it is often part of its clinical presentation (5–7, 10, 11). Indeed, Jensen et al. (2007) reported that more than 50% of preadolescents with ADHD exhibit clinically significant aggression (10). Importantly, impulsive aggressive behaviour is a strong predictor of a highly impaired developmental trajectory, associated with greater persistence of ADHD symptomatology throughout life, as well as more severe functional deficits, greater psychosocial impairment, and higher prevalence of comorbidities (7). However, the causes of impulsive aggressive behaviour can be multiple and vary between individuals.

Executive function (EF) deficits, such as poor inhibitory control, are common in children with ADHD (12, 13) and may predispose them to impulsive and reckless behaviour, contributing to aggressive and externalising behaviours (14, 15). Inhibitory control (or response inhibition) is an executive function broadly conceptualised as the individual’s ability to inhibit or suppress impulses and natural, dominant, or prepotent inappropriate responses to stimuli (16). This ability is crucial for the selection and execution of what is more appropriate or needed, when the context demands it (16). The failure to inhibit or suppress such prepotent responses in selecting goal-directed and appropriate behaviours likely generates aggression through frustration (17–19). Moreover, inhibitory control is intrinsically implicated in self-regulatory skills (20–22). Indeed, as Barkley (23, 24) proposed in his theoretical approach, executive functioning and its underlying brain networks are the foundations for self-regulation and, accordingly, ADHD is a self-regulation deficit disorder.

Self-regulation refers to processes that serve to modulate reactivity, including the child’s ability to modulate their thoughts, emotions, and behaviours, in order to adapt and adjust to different situations effectively (25). Accordingly, it is the repeated failure to demonstrate self-regulation that is evident in ADHD children, and deficits in executive function underlie that phenotype (24). Furthermore, inhibitory control is associated not only with suppressing or regulating behaviours but also with emotion (dys)regulation (26). Not surprisingly, more recently, impulsive aggression has also been attributed to emotion dysregulation (27, 28), which is argued to be a core feature of ADHD (23, 24). Emerging studies with typically developing children, along with conceptual models, suggest that well-developed inhibitory control may support the child’s ability to regulate emotions, and, on the contrary, deficits in inhibitory control may contribute to emotion dysregulation (29, 30). However, empirical studies showing this relationship in children with ADHD are scarce (31). Emotional symptoms are frequently manifested by children with ADHD – which is why some consider them to be a core feature rather than an associated trait – and are linked to increased morbidity (32). Remarkably, 40 to 50% of children with ADHD have significant impairments in regulating their emotions, easily showing manifestations that stem from anger, irritability, or frustration (33–35).

Emotion dysregulation refers to patterns of emotional experience and expression wherein individuals are unable to modify their emotional states, thereby hindering their ability to engage in adaptive behaviours conducive to achieving their goals (36–38). Emotion dysregulation involves multiple and complex domains of the emotion generation process, such as emotion recognition/understanding, emotion reactivity/negativity/lability, emotion regulation, and empathy/callous-unemotional traits (39). According to the meta-analytic study conducted by Graziano & Garcia (2016), the greatest impairment among youth with ADHD is observed in emotion reactivity/negativity/lability and emotion regulation (39). Although intrinsically related, emotion regulation and emotional lability are separate constructs (40), with emotion regulation referring to children’s ability to control and modify extreme states of emotional or behavioural arousal in such a way that reciprocal and rewarding social interactions become possible (40). Emotional lability refers to the difficulty and/or inability to maintain stable and consistent emotional states over time (41).

Emotion dysregulation reflects maladaptive mechanisms by which individuals experience and respond to emotional states (42) and is currently considered a defining feature of many forms of psychopathology, both internalising or externalising (43–47). A longitudinal study, involving 1065 adolescents between 11 to 14 years old, found that baseline emotion dysregulation predicted increases in symptoms of anxiety, aggressive behaviour and eating disorders over time (48). Importantly, they also found that baseline psychopathology is not a predictor of changes in emotion dysregulation across time (48). In fact, emotion dysregulation is currently assumed as a transdiagnostic factor of psychopathology (43, 49, 50), as most forms of psychopathology imply a non-adaptive experience of emotions (51, 52). Within ADHD, emotion dysregulation predicts the persistence of the disorder over time, but also the development of aggressive behaviours (53). Indeed, emotion dysregulation tends to increase its expression across development among those with ADHD that follow a developmental trajectory into more severe externalising behaviour (54, 55). According to the developmental psychopathology framework (i) ADHD may act as a risk factor for the development of other conditions, (ii) shared risk factors between conditions can contribute to the simultaneous occurrence of ADHD and comorbidities, and (iii) correlates or consequences of ADHD constitute risk for the co-occurrence of other conditions (56, 57). This theoretical framework gives conceptual support both to the co-occurrence of aggressive problems within ADHD, but also to the development of depressive symptoms, where emotion dysregulation may act as a shared risk process (57). As seen previously, some empirical evidence exists for emotion dysregulation as a predictor of the development of aggressive behaviours within ADHD (53), and also some support for emotion dysregulation as a potential mechanism linking ADHD and depressive symptoms (58).

Continuous depressive symptoms (as opposed to isolated major depressive episodes) are manifested in higher levels in youth with ADHD, when compared to their peers without the disorder (59–63). Additionally, this population is also at a higher likelihood of being diagnosed with a mood disorder, such as major depressive disorder, before age 18 (64). However, when both co-occur during childhood or adolescence, the ADHD diagnosis often precedes the one of major depressive disorder, so there must be correlates of ADHD contributing to the onset of depression (57). Emotion regulation difficulties in depressive children might be expressed through high levels of negative affectivity, increased intensity of negative emotions, emotional lability, and poor effortful control (65–67). The inability to disengage from negative affect and thoughts (e.g., rumination) is particularly common in depression (45) and is hypothesised to be due to low inhibitory control (67). On the other hand, children with poor self-regulation may cope with negative emotions and thoughts through aggressive and impulsive behaviours (68). Although not well established, the relationship between depressive symptoms and aggressive behaviour finds some support in the ADHD literature. In fact, aggressive boys with ADHD express higher levels of depressive symptoms than nonaggressive boys with ADHD, who, in turn, reveal more depressive symptoms than non-ADHD boys (69).

While there is general support for direct relationships between these key variables, to our knowledge, no studies have examined whether emotion dysregulation and depressive symptoms mediate the association between deficits in inhibitory control and impulsive aggressive behaviour in ADHD. Thus, the current study aimed to (i) compare ADHD and typically developing (TD) Portuguese children regarding parent-reported aggressive behaviour, hypothesising that this type of externalising problem is more frequent in children with ADHD, supporting aggression as part of the clinical presentation of the disorder (6) and (ii) investigate whether emotion dysregulation (in the domains of emotional lability and emotion regulation) and depressive symptoms account for (i.e., mediate) the relationship between inhibitory control difficulties and aggression problems in children with ADHD. We hypothesise that higher deficits in inhibitory control will be associated with higher expression of aggression, as others had previously suggested (14, 15). Moreover, emotion dysregulation and depressive symptoms are expected to mediate the association between inhibitory control difficulties and aggressive behaviour. This is suggested by theoretical models (23, 57) and previous empirical research supporting this association (28, 67, 70, 71). Specifically, we hypothesise that higher emotion dysregulation and depressive symptoms are expected to at least partially account for the association between deficits in inhibitory control and aggressive behaviour.

## Methods

### Study design and procedure

This study was conducted in Portugal. The data were derived from a larger case-control study designed to investigate the neurobiological mechanisms underlying ADHD. Recruitment and data collection occurred between January 2021 and December 2022. Participants were recruited from the Department of Paediatrics of the University Hospital Centre of São João (CHUSJ), and the community through word of mouth and web advertisement. Written informed consent was obtained from the accompanying parent(s), usually the mother, who then completed the questionnaires under a research assistant’s surveillance. The broader study from which data for this study were derived was approved by the Ethics Committee of CHUSJ in Portugal (318/2020).

### Participants

Seventy-two children between the ages of 6 and 10 years, and without neurological disorders, traumatic brain injury, intellectual disability, and known genetic diseases, were recruited. Thirty-eight had received a prior diagnosis of ADHD by a neurodevelopmental paediatrician according to clinical criteria established by the Diagnostic and Statistical Manual of Mental Disorders 5th Edition (DSM-5) (72) and standardised questionnaires, specifically the Conners’ Rating Scales-Revised (CRS-R; Conners, 1997; Portuguese adaptation by Rodrigues, 2000). Thirty-four were typically developing (TD). At study enrolment, the Conners’ Parent Rating Scale-Revised: Short Form (CPRS-R:S) was used to confirm/rule out ADHD symptomatology. The majority of ADHD children were referred to us by CHUSJ and underwent screening for psychiatric comorbidities, such as Oppositional Defiant Disorder (ODD) and Autism Spectrum Disorder (ASD). Those with identified comorbidities were not included in the referral process. The remaining children were recruited from the community without prior screening, but psychiatric comorbidities were determined via medical records and/or parent report. Children with reported ODD and/or ASD were excluded from this study. Socioeconomic status was derived from parent(s)’ profession, level of schooling, sources of family income, comfort of housing and place of residence via the administration of the Graffar Scale to the accompanying parent(s).

### Measures

#### CPRS-R:S

The Conners’ Parent Rating Scale-Revised: Short Form (CPRS-R:S; Conners, 1997; Portuguese adaptation by Rodrigues, 2000) assesses childhood behaviour problems and assists in evaluating children and adolescents for ADHD. The CPRS-R:S consists of 27 items to which parents assign a score based on a four-point Likert scale indicating how frequent each item is for their child regarding the past month (0 = ‘Never’, 1 = ‘Sometimes’, 2 = ‘Often’, 3 = ‘Very often’), with higher scores representing more severe problems. Raw scores are converted to T-scores, weighed by sex and age. It is possible to obtain three scales (‘Oppositional’, ‘Cognitive problems/Inattention’, and ‘Hyperactivity’) and the ADHD Index, in which a T-score < 55 is average (typical), between 56 and 60 is slightly atypical (borderline), between 61 and 65 is mildly atypical (possible problem), between 66 and 70 is moderately atypical (significant problem), and a T-score > 70 is markedly atypical (significant problem). In the Portuguese population, no normative data could be derived for the ‘Oppositional’ scale; therefore, only the ‘Cognitive problems/Inattention’ and ‘Hyperactivity’ scales and the ADHD Index T-scores will be reported. Construct reliability was assessed using Cronbach’s alpha and results revealed that the ‘Cognitive problems/Inattention’ scale (5 items, α = .938), the ‘Hyperactivity’ scale (7 items, α = .940), and the ADHD Index (12 items, α = .954) were all found to be reliable.

#### CBCL/6-18

The Child Behaviour Checklist (CBCL; Achenbach, 2001; Portuguese adaptation by Gonçalves, Dias, & Machado, 2007), a component of the Achenbach System of Empirically Based Assessment (ASEBA), assesses children and adolescents’ behavioural and emotional problems. The version used in this study – CBCL/6-18 – consists of 113 items to which parents assign a score based on a three-point Likert scale indicating how true each item is for their child regarding the past six months (0 = ’Not true’, 1 = ’Somewhat or sometimes true’, 2 = ’Very true or often true’), with higher scores representing more severe problems. Raw scores are converted to T-scores, weighted by sex and age. It is possible to obtain scores for three main scales (‘Internalising problems’, ‘Externalising problems’, and ‘Total problems’), six scales based on the DSM-IV (‘Affective problems’, ‘Anxiety problems’, ‘Somatic problems’, ‘Attention Deficit/Hyperactivity Disorder problems’, ‘Oppositional Defiant problems’, and ‘Conduct problems’), and eight empirically-based syndrome scales (‘Anxious/Depressed’, ‘Withdrawn/Depressed’, ‘Somatic complaints’, ‘Social problems’, ‘Attention problems’, ‘Rule-breaking behaviour’, and ‘Aggressive behaviour’) in which a T-score ≤ 64 indicates non-clinical symptoms, a T-score between 65 and 69 indicates a borderline range, and a T-score ≥ 70 indicates clinical symptoms. For the present analysis, only the ‘Anxious/Depressed’, ‘Withdrawn/Depressed’ and ‘Aggressive behaviour” T-scores will be considered. Construct reliability was assessed using Cronbach’s alpha and results revealed that the ‘Anxious/Depressed’ (13 items, α = .825), ‘Withdrawn/Depressed’ (8 items, α = .763) and ‘Aggressive behaviour” (18 items, α = .924) scales were found to be reliable.

#### BRIEF – Parent Form

The Behavior Rating Inventory of Executive Function (BRIEF; Gioia et al., 2000) – Parent Form – assesses executive function behaviours at home and at school in children and adolescents. The BRIEF – Parent Form – consists of 86 items to which parents assign a score based on a three-point Likert scale indicating how frequent each item is for their child regarding the past six months (1 = ‘Never’, 2 = ‘Sometimes’, 3 = ‘Often’), with higher scores representing more severe dysfunction. It is possible to obtain two broad indexes (‘Behavior Regulation Index’ and ‘Metacognitive Index’) and an overall score (‘Global Executive Composite’). The ‘Behavior Regulation Index’ consists of three clinical scales (‘Inhibit’, ‘Shift’, and ‘Emotional Control’), and the ‘Metacognition Index’ of five clinical scales (‘Initiate’, ‘Working Memory’, ‘Plan/Organise’, ‘Organisation of materials’, and ‘Monitor’). Raw scores were used in this study as T-scores are not yet available for the Portuguese population. For the present analysis, only the ‘Inhibit’ and ‘Emotional Control’ raw scores will be considered. Construct reliability was assessed using Cronbach’s alpha and results revealed that the ‘Inhibit’ (10 items, α = .919) and ‘Emotional Control’ (10 items, α = .942) clinical scales were found to be reliable.

#### ERC

The Emotion Regulation Checklist (ERC; Shields & Cichetti, 1997; Portuguese version by Melo & Soares, 2005) assesses children’s self-regulation. The ERC consists of 24 items to which parents assign a score based on a four-point Likert scale indicating how frequent each item is for their child (1 = ‘Never’, 2 = ‘Sometimes’, 3 = ‘Often’, 4 = ‘Always’). Two scales are obtained: ‘Lability/Negativity’ and ‘Emotion Regulation’. The ‘Lability/Negativity’ scale assesses lack of flexibility, anger dysregulation, and mood lability, with higher scores representing greater emotion dysregulation; while the ‘Emotion Regulation’ scale assesses expression of emotions, empathy, and emotional self-awareness, with higher scores representing greater adaptive regulatory processes. Raw scores were used in this study as T-scores are not yet available for the Portuguese population. As a result, for the present analysis, the ‘Lability/Negativity’ and ‘Emotion Regulation’ raw scores will be considered. Construct reliability was assessed using Cronbach’s alpha and results revealed that the ‘Lability/Negativity’ (15 items, α = .894) and ‘Emotion Regulation’ (8 items, α = .728) scales were found to be reliable.

### Statistical analyses

All statistical analyses were conducted using IBM SPSS Statistics 29 (RRID : SCR_019096). The normality of data distribution was assessed using the Shapiro-Wilk test. The differences in study variables between children with ADHD and TD children were investigated using the Mann-Whitney U test for non-normally distributed data, and the independent samples T-test for normally distributed data. The chi-square test was used for categorical variables. A *p*-value of less than .05 was considered statistically significant. Next, we conducted factor analyses using the principal axis factoring method to distil variables related to emotional (dys)regulation into a single factor. The principal axis factoring method was used given its suitability for identifying a single dominant factor, its robustness for non-normally distributed data, and its appropriateness for smaller sample sizes. The resulting composite score was computed by combining the scores of the variables, weighted by their respective factor loadings, providing a succinct measure of emotion dysregulation. The relationships between all study variables, as well as the emotion dysregulation composite score, and potential covariates were subsequently assessed using Spearman’s rank correlation coefficient for non-normally distributed data, and the Pearson correlation coefficient for normally distributed data. Then, to check that all regression assumptions (linearity of the relationships between the dependent variable and the independent variable(s), normal distribution of residuals, homoscedasticity of the residuals, uncorrelatedness of residuals, absence of strong multicollinearity, appropriate scale properties, and absence of extreme outliers) were met before testing mediation models, we performed regression models, based on directions provided by Regorz (73). The normality of residuals was checked using the Shapiro-Wilk test and by visually inspecting QQ plots. No issues were identified. Finally, to test our hypotheses, we conducted mediation analyses using Hayes’ PROCESS macro (version 4.2; RRID : SCR_021369) for IBM SPSS Statistics. According to Fritz & Mackinnon (74), when using a percentile bootstrap test, which is the default in PROCESS version 4, a sample size of 36 is required for large effect sizes in both the a-path and the b-path and a power of .80. Therefore, the current study’s sample size of 38 children with ADHD is sufficient to achieve adequate power. Although no issues were detected when checking regression assumptions, we used all possible methods to ensure no violation of assumptions when testing all mediation models, including 5,000 bootstrap samples (with 95% confidence intervals) and a robust standard error (HC4), for heteroscedasticity-consistent inference. Indirect effects were considered significant if the confidence interval did not include 0.

## Results

### Participant characterisation

Descriptive statistics were calculated for the entire sample, and participants’ demographic characterisation and clinical characterisation are detailed in **Tables 1**, **2**, respectively. The 72 children, ranging from 6 to 10 years old, consisted of 38 ADHD and 34 TD. The ADHD group included 30 males and 8 females; the median age was 9 years (IQR 7-10) and the median age in months was 109 (IQR 92-122). The TD group included 16 males and 18 females; the median age was 8 years (IQR 7-9) and the median age in months was 102.50 (IQR 89-117). In the ADHD group, 74% were classified as upper middle to upper class, 24% as middle class, and 1% as low to lower middle class. Ninety-four percent of the TD group were classified as upper middle to upper class, and 6% as middle class. Seventy-four percent of participants with ADHD were taking ADHD medication. Within the ADHD group 14% had received a diagnosis of Specific Learning Disorder (SLD). TD children had no known psychiatric disorders except for one case (3%) who had received a SLD diagnosis. All TD children had non-clinical scores on CPRS-R:S Cognitive problems/Inattention and Hyperactivity scales and ADHD Index.

**Table 1:** Participants’ demographic characterisation (*n* = 72).

	ADHD (n = 38)	TD (n = 34)	*p*
Median (IQR)			
Age			
Years	9 (7-10)	8 (7-9)	.548
Total in months	109 (92-122)	102.50 (89-117)	.349
N (%)			
**Sex**			.007
Male	30 (78.95)	16 (47.06)	
Female	8 (21.05)	18 (52.94)	
**Socioeconomic status**			.020
Upper middle to upper class	28 (73.68)	32 (94.12)	
Middle class	9 (23.68)	2 (5.88)	
Low to lower middle class	1 (2.63)	0 (0)	

**Table 2:** Participants’ clinical characterisation (*n* = 72).

	ADHD (n = 38)	TD (n = 34)	*p*
N (%)			
**Medication**			<.001
ADHD medication	28 (73.68)	0 (0)	
**Common disorders/comorbidities**			.007
Specific Learning Disorder	6 (15.79)	1 (2.94)	
Others^a^	6 (15.79)	0 (0)	
Median (IQR)			
CPRS-R:S^b^			
Cognitive problems/Inattention	69 (63-76)	40 (39-54)	<.001
Hyperactivity	71.50 (58-77)	43.50 (41-51)	<.001
ADHD Index	70 (66-75)	42 (38-54)	<.001
CBCL/6-18^b^			
Anxious/Depressed	62 (57-67)	51.50 (50-62)	<.001
Withdrawn/Depressed	66 (62-70)	54 (50-58)	<.001
Aggressive behaviour	65 (61-72)	52 (50-56)	<.001
BRIEF – Parent Form^c^			
Inhibit	22 (19-24)	13 (11-17)	<.001
Emotional Control	21 (18-27)	12 (11-17)	<.001
ERC^c^			
Lability/Negativity	25 (21-29)	16 (14-21)	<.001
Emotion Regulation	18.50 (16-20)	20.50 (18-22)	.013

a Includes: Global Developmental Delay (n = 2), Communication Disorder (n = 2), Motor disorders (n = 2).

b T-scores.

c Raw scores.

CPRS-R:S, Conners’ Parent Rating Scale–Revised: Short Form; CBCL/6-18, Child Behaviour Checklist for Ages 6-18; BRIEF, Behaviour Rating Inventory of Executive Function; ERC, Emotion Regulation Checklist.

### Differences in study variables between ADHD and TD children

Significant differences were found between ADHD and TD children for all study variables, namely depressive symptoms, inhibitory control difficulties, emotional control difficulties, emotional lability, and emotion regulation (see **Table 2**). Regarding our outcome variable – aggressive behaviour – children with ADHD had significantly higher scores (median 65, IQR 61-72) compared to TD children (median 52, IQR 50-56), U = 202.500, Z = 5.022, *p* = <.001, r = .592.

### Factor analysis and constructing an emotion dysregulation composite score

To identify a single factor representing emotion dysregulation, we conducted a factor analysis using the principal axis factoring method. The analysis incorporated three variables related to emotional (dys)regulation: BRIEF Emotional Control, ERC Lability/Negativity and ERC Emotion Regulation. The analysis revealed a single factor with an eigenvalue of 2.10, explaining 70.08% of the variance. The Keiser-Meyer-Olkin measure of sampling adequacy was .573, which is considered satisfactory. Bartlett’s Test of Sphericity was significant, χ^2^(n = 72) = 109.950 (*p* = <.001). The determinant score was .204, which is considered adequate. However, due to poor factor loading and communalities under .5, ERC Emotion Regulation was excluded from the factor analysis, resulting in a notable enhancement in the explanatory power of the derived factor. The revised analysis, excluding ERC Emotion Regulation, demonstrated a substantial increase in the variance explained, rising from 70.08% to 93.70%, with an eigenvalue of 1.87. The Keiser-Meyer-Olkin measure of sampling adequacy was .500, considered satisfactory. Bartlett’s Test of Sphericity was significant, χ^2^(n = 72) = 100.308 (*p* = <.001). The determinant score was .236, considered adequate. Factor loadings for BRIEF Emotional Control and ERC Lability/Negativity remained robust (above .9), contributing significantly to the derived factor. This decision was made to optimise the clarity and coherence of the extracted factor since the poor factor loading of ERC Emotion Regulation suggested its limited contribution to the coherent representation of emotion dysregulation in this particular sample. Subsequently, we computed a composite score by summing the products of each variable’s score and its corresponding factor loading obtained from the factor matrix. This approach ensures that the composite score gives more weight to variables with higher factor loadings, providing a refined measure of emotion dysregulation based on the selected variables.

Differences in the Emotion Dysregulation composite score between children with ADHD and TD children were investigated using the Mann-Whitney U test. Children with ADHD had significantly higher scores (median 42.96, IQR 36-51) compared to TD children (median 25.69, IQR 23-35), U = 157, Z = 5.520, *p* = <.001, r = .651.

### Mediation models in ADHD children

Hereafter, only the ADHD children (n = 38) will be considered. Correlations were calculated between all study variables, as well as the emotion dysregulation composite score derived from the abovementioned factor analyses, and potential covariates (age, sex, socioeconomic status, and medication status) and are indicated in **Table 3**. Because the CBCL Anxious/Depressed scale showed no or weak correlations with the other study variables it was excluded from the mediation analyses. Given that no potential covariates (namely age, sex, socioeconomic status, and medication status) had significant correlations with any of the study variables, they were not controlled for in any of the mediation models.

**Table 3:** Correlations between potential covariates and study variables in ADHD children (n = 38).

	1	2	3	4	5	6	7	8	9	10	11	12
1. Age	–											
2. Sex^a^	-.156	–										
3. Socioeconomic status	.142	-.165	–									
4. Medication status^b^	-.188	.162	.057	–								
5. CBCL: Anxious/Depressed	-.052	-.109	.268	.213	_							
6. CBCL: Withdrawn/Depressed	.047	-.146	.048	-.187	.138	_						
7. CBCL: Aggressive behaviour	.010	-.009	.017	-.090	.268	.690^***^	_					
8. BRIEF: Inhibit	.009	-.098	.212	-.014	.153	.454^**^	.619^***^	_				
9. BRIEF: Emotional Control	.036	-.071	.034	.057	.334^*^	.488^**^	.718^***^	.625^***^	_			
10. ERC: Lability/Negativity	.168	.068	-.098	.036	.217	.620^***^	.752^***^	.678^***^	.785^***^	_		
11. ERC: Emotion Regulation	-.397	.116	-.248	.008	-.238	-.232	-.390^*^	-.302	-.291	-.345^*^	_	
12. Emotion dysregulation composite score^c^	.115	-.018	-.032	.046	.291	.587^***^	.778^***^	.690^***^	.944^***^	.945^***^	-.337^*^	_

a Sex, 0 = male, 1 = female.

b edication status, 0 = no ADHD medication taken, 1 = ADHD medication taken.

c Computed by summing the products of each variable’s score and its corresponding factor loading obtained from the factor matrix.

CBCL, Child Behaviour Checklist for Ages 6-18; BRIEF, Behaviour Rating Inventory of Executive Function – Parent Form; ERC, Emotion Regulation Checklist.

* p <.05. ** p <.01. *** p <.001.

#### Simple mediation

**Figure 1:**
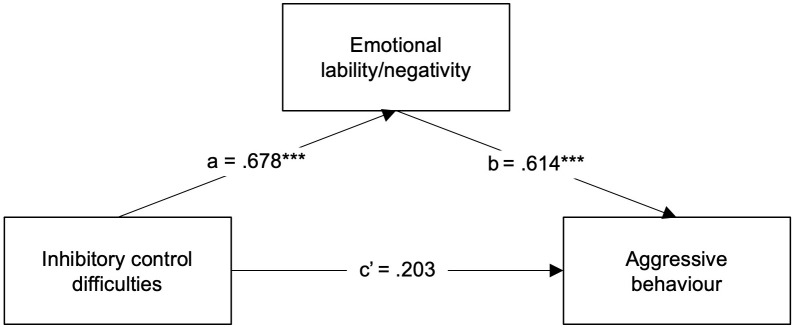
Regression coefficients (*β*) showing the relationship between inhibitory control difficulties and aggressive behaviour via emotional lability/negativity in ADHD children (n = 38). Standardised coefficients are displayed. ***p <.001.

To explore whether emotional lability/negativity acts as a mediator in the association between inhibitory control difficulties and aggressive behaviour in ADHD, we tested a simple mediation model (model 4). The BRIEF Inhibit clinical scale was entered as the predictor variable (X), the ERC Lability/Negativity scale was entered as the mediator (M), and the CBCL Aggressive behaviour scale as the outcome variable (Y). Parent-reported inhibitory control difficulties were significantly associated with parent-reported aggressive behaviour (total effect *β* = .619, *p* = .000, 95% CI [.888, 2.065) and with parent-reported emotional lability/negativity (a path *β* = .678, *p* = .000, 95% CI [.543, 1.345]). The association between emotional lability/negativity and aggressive behaviour was also significant (b path *β* =.614, *p* = .000, 95% CI [.611, 1.494]). When emotional lability/negativity was introduced as a mediator (**Figure 1**), the direct association between aggressive behaviour and inhibitory control difficulties was no longer significant (direct effect *β* = .203, *p* = .132, 95% CI [-.153, 1.119]), while the indirect association was significant and positive (indirect effect *β* = .417, 95% CI [.220,.620]). Thus, the association between inhibitory control difficulties and aggressive behaviour in ADHD children seems to be mediated by emotional lability/negativity. This simple mediation model explained 58.7% of the variance in aggressive behaviour (R^2^ = .587, *F (2*, 35) = 24.344, *p* = .000).

**Figure 2:**
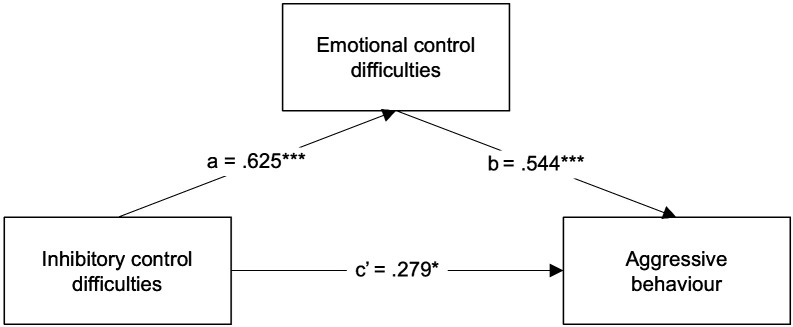
Regression coefficients (*β*) showing the relationship between inhibitory control difficulties and aggressive behaviour via emotional control difficulties in ADHD children (n = 38). Standardised coefficients are displayed. *p <.05, ***p <.001.

Next, to explore whether emotional control difficulties act as a mediator in the association between inhibitory control difficulties and aggressive behaviour in ADHD, the BRIEF Emotional Control clinical scale was entered instead as the mediator (M) in a new simple mediation model. Parent-reported inhibitory control difficulties were significantly associated with parent-reported emotional control difficulties (a path *β* = .625, *p* = .000, 95% CI [.538, 1.190]). The association between emotional control difficulties and aggressive behaviour was also significant (b path *β* =.544, *p* = .000, 95% CI [.456, 1.421]). When emotional control difficulties were introduced as a mediator (**Figure 2**), though the indirect association was significant and positive (indirect effect *β* = .340, 95% CI [.170,.546]), the direct association between aggressive behaviour and inhibitory control difficulties was still significant (direct effect *β* = .279, *p* = .034, 95% CI [.052, 1.279]), Thus, the association between inhibitory control difficulties and aggressive behaviour in ADHD children seems only to be partially mediated by emotional control difficulties. This simple mediation model explained 56.3% of the variance in aggressive behaviour (R^2^ = .563, *F (2*, 35) = 25.951, *p* = .000), which, alongside its partial mediation status, showcases the decreased explanatory power of emotional control difficulties in this model.

**Figure 3:**
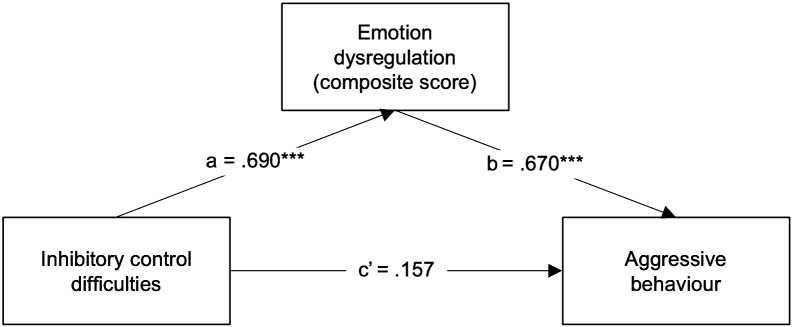
Regression coefficients (*β*) showing the relationship between inhibitory control difficulties and aggressive behaviour via emotion dysregulation (derived composite score) in ADHD children (n = 38). Standardised coefficients are displayed. ***p <.001.

Then, to explore whether the composite score representing emotion dysregulation could best explain the association between inhibitory control difficulties and aggressive behaviour in ADHD, the Emotion Dysregulation composite score was entered instead as the mediator (M) in a new simple mediation model. Parent-reported inhibitory control difficulties were significantly associated with parent-reported emotion dysregulation (a path *β* = .690, *p* = .000, 95% CI [1.057, 2.231]). The association between emotion dysregulation and aggressive behaviour was also significant (b path *β* =.670, *p* = .000, 95% CI [.401,.905]). When emotion dysregulation was introduced as a mediator (**Figure 3**), the direct association between aggressive behaviour and inhibitory control difficulties was no longer significant (direct effect *β* = .157, *p* = .236, 95% CI [-.256, 1.004]), while the indirect association was significant and positive (indirect effect *β* = .462, 95% CI [.254,.687]). Thus, the association between inhibitory control difficulties and aggressive behaviour in ADHD children seems to be mediated by emotion dysregulation. This simple mediation model explained 61.8% of the variance in aggressive behaviour (R^2^ = .593, *F (2*, 40) = 31.490, *p* = .000), demonstrating a 3.1% and 5.5% increase in variance explained compared to the previous two simple mediation models.

**Figure 4:**
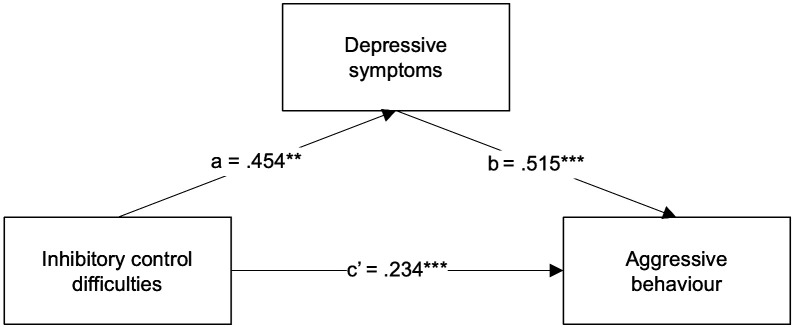
Regression coefficients (*β*) showing the relationship between inhibitory control difficulties and aggressive behaviour via depressive symptoms in ADHD children (n = 38). Standardised coefficients are displayed. **p < 0.01; ***p < 0.001.

Finally, to explore whether depressive symptoms act as a mediator in the association between inhibitory control difficulties and aggressive behaviour in ADHD, we tested a simple mediation model (model 4). The BRIEF Inhibit clinical scale was entered as the predictor variable (X), the CBCL Withdrawn/Depressed scale was entered as the mediator (M), and the CBCL Aggressive behaviour scale as the outcome variable (Y). Parent-reported inhibitory control difficulties were significantly associated with parent-reported depressive symptoms (a path *β* = .454, *p* = .003, 95% CI [.292, 1.326]). The association between depressive symptoms and aggressive behaviour was also significant (b path *β* =.515, *p* = .000, 95% CI [.479,.899]). When depressive symptoms were introduced as a mediator (**Figure 4**), though the indirect association was significant and positive (indirect effect *β* = .234, 95% CI [.066,.394]), the direct association between aggressive behaviour and inhibitory control difficulties was still significant (direct effect *β* = .385, *p* = .000, 95% CI [.435, 1.403]). Thus, the association between inhibitory control difficulties and aggressive behaviour in ADHD children seems only to be partially mediated by depressive symptoms. This simple mediation model explained 59.4% of the variance in aggressive behaviour (R^2^ = .594, *F*(2, 35) = 42.558, *p* = .000). It is worth mentioning, however, that the indirect association was nearly non-significant, as the confidence interval nearly included 0.

#### Serial mediation

**Figure 5:**
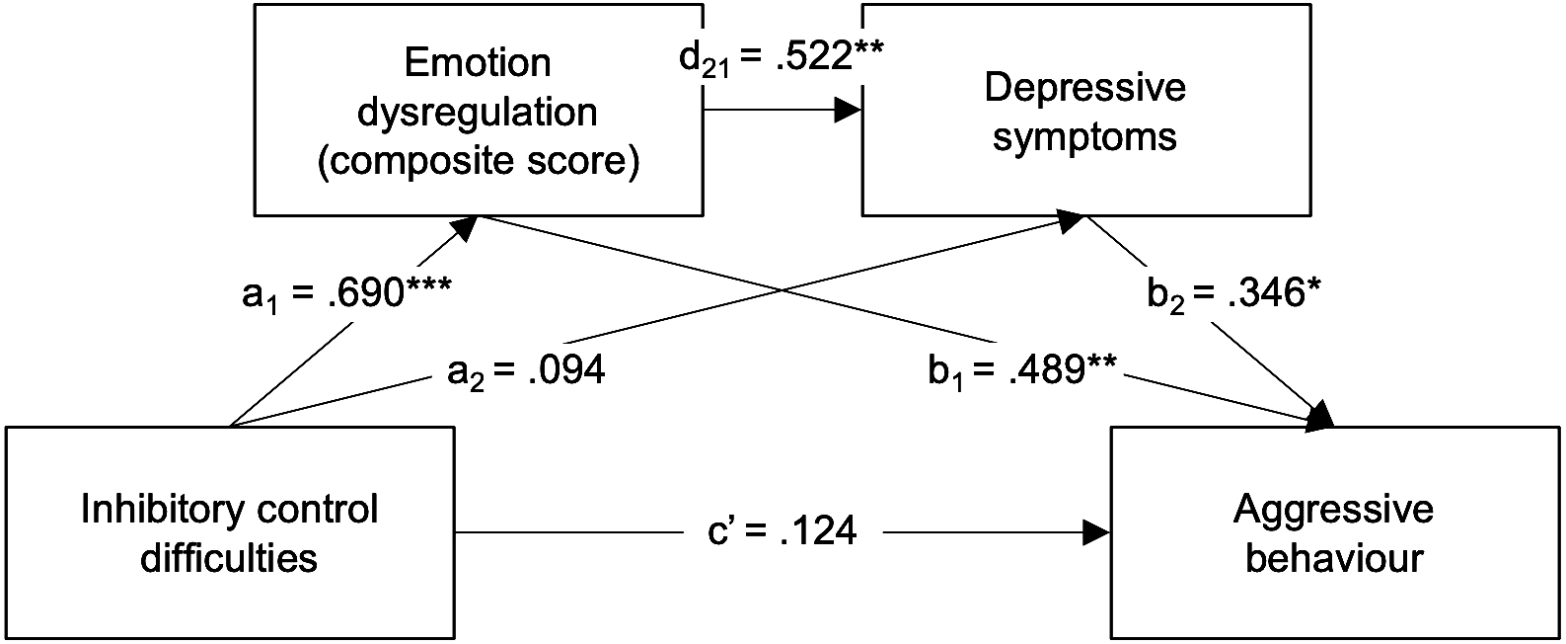
Regression coefficients (*β*) showing the relationship between inhibitory control difficulties and aggressive behaviour via emotion dysregulation (derived composite score) and depressive symptoms in ADHD children (n = 38). Standardised coefficients are displayed. *p <.05, **p <.01; ***p <.001.

Although depressive symptoms did not fully mediate the relationship between inhibitory control difficulties and aggressive behaviour, we observed depressive symptoms were significantly correlated with both emotion dysregulation and aggressive behaviour (see **Table 3**). Considering these findings, along with the theoretical framework presented, and given that the emotion dysregulation composite score appeared to best explain the association between inhibitory control difficulties and aggressive behaviour in ADHD, we then explored whether emotion dysregulation and depressive symptoms act as serial mediators in the association between inhibitory control difficulties and aggressive behaviour in ADHD by testing a serial mediation model (model 6). Here, the Emotion Dysregulation composite score was entered as the first mediator (M1) and the CBCL Withdrawn/Depressed scale as the second mediator (M2). When parent-reported depressive symptoms were introduced to the first model as a second mediator (M2) (**Figure 5**), the direct association between inhibitory control difficulties and aggressive behaviour was reduced compared to the simple mediation model and remained non-significant (direct effect *β* = .124, *p* = .298, 95% CI [-.274,.867]); while the third indirect effect (X-M1-M2-Y) was significant and positive (indirect effect *β* = .125, 95% CI [.020,.270]). Thus, the association between inhibitory control difficulties and aggressive behaviour in ADHD children seems to be mediated by emotion dysregulation affecting depressive symptoms, which in turn mediates aggressive behaviour. That is, inhibitory control difficulties predicted greater emotion dysregulation, which predicted greater depressive symptoms, which, in turn, predicted greater levels of aggressive behaviour. The simple indirect effect of inhibitory control difficulties on aggressive behaviour via depressive symptoms (X-M2-Y) was not significant (indirect effect *β* = .033, 95% CI [-.107,.188). This serial mediation model explained 69.6% of the variance in aggressive behaviour (R^2^ = .696, *F*(3, 34) = 22.180, *p* = .000), which reveals that depressive symptoms play a relevant part in this model. It also highlights the synergistic role of emotion dysregulation and depressive symptoms in the relationship between inhibitory control difficulties and aggressive behaviour.

## Discussion

The current study aimed to (i) compare ADHD with typically developing (TD) Portuguese children regarding parent-reported aggressive behaviour, and (ii) elucidate the associations between inhibitory control difficulties, emotion dysregulation, depressive symptoms and aggressive behaviour in a clinical sample of children diagnosed with ADHD. The findings suggest that children with ADHD show higher levels of aggressive behaviour and that the relationship between inhibitory control and aggressive behaviour is fully mediated by emotion dysregulation and depressive symptoms.

The results from this study support previous findings demonstrating that aggression problems, although not diagnostic, are common in ADHD children (5–7, 10), and significantly more frequent in ADHD children when compared with non-ADHD children (75). This result also seems in line with and corroborates one of the developmental psychopathology framework assumptions that ADHD acts as a risk factor for the development of other co-occurring problems (57). Indeed, aggressive behaviours often emerge in normative development as a manifestation of the child’s anger, but they tend to decrease as the child develops their cognitive, language and emotion regulation abilities (8). It is not a marker of a specific diagnosis but could be used as a sensitive indicator of severity of psychopathology, as there is a substantial surge in clinical (*versus* nonclinical) samples (10). Impulsive aggression (in contrast to planned/instrumental aggression) was the type of aggression measured in our study, as it seems to be the type primarily manifested by ADHD children (10). By definition, impulsive aggression is unplanned and overt, and the perpetrator does not anticipate the outcome of the aggressive act, which is then perceived to be negative (76). This process of acting out without anticipating consequences is accompanied by negative emotions, such as frustration, regret, guilt, and fear (76). This is in line with our study’s second aim, which was to investigate whether emotion dysregulation and depressive symptoms account for (i.e., mediate) the relationship between inhibitory control difficulties and aggressive behaviour in a clinical sample of children diagnosed with ADHD. As predicted, there was a significant direct effect of inhibitory control difficulties on aggressive behaviour, which is fully mediated by both emotion dysregulation and depressive symptoms; that is, inhibitory control impairments were associated with emotion dysregulation, which was associated with higher depressive symptoms, which in turn was associated with higher levels of aggressive behaviour. This finding directly supports components of the theoretical model proposed by Barkley (23), which conceptualises the impulsive trait of ADHD children as a deficit in self-regulation that stems from executive inhibitory control deficits. This specific deficit may predispose ADHD children to impulsive and reckless behaviour, contributing to aggressive and externalising problems (14, 15).

Previous studies show that inhibitory control, being the capacity to inhibit a dominant action, tends to increase throughout development, with significant changes observed between the preschool and first formal school years (15). However, in ADHD children, the maturation of this cognitive ability is compromised. Thus, by school age, these children tend to manifest a delay in their ability to inhibit their prompt and unplanned responses (77). Consequently, impulsive aggression is likely when real or perceived provocations emerge in their environment. Additionally, impulsive aggression is argued to be a response that reflects out-of-control emotionality mitigating immediate emotional burden (7), which is supported by our results showing that emotion dysregulation plays a key role in aggressive behaviour in ADHD children (7).

The main result of our study supports the central role of emotion dysregulation in ADHD, suggesting it as a shared risk factor between ADHD and aggressive behaviours, as proposed by the developmental psychopathology approach to ADHD and comorbidities (57), underlying the important link between inhibitory control difficulties and impulsive aggressive behaviours. As Barkley proposed (33), and which our results corroborate, emotion dysregulation is considered to be a consequence of deficient executive inhibitory control (31). Therefore, the extent to which a child with ADHD expresses deficits in behavioural inhibition is the extent to which they will automatically display a corresponding degree of difficulty in emotional inhibition (33).

Importantly, our results also highlight the higher levels of depressive symptoms in children with ADHD when compared to typically developing pairs. As previously noted, continuous depressive symptoms are more evident in children with the disorder (60–63), suggesting that there are correlates of ADHD that contribute to the emergence of these symptoms (57). Possible correlates were described in the introduction section but will not be discussed here as they are beyond the scope of this article. Furthermore, our results also highlight the role of depressive symptoms in the mediation model, corroborating what has been reported regarding emotional difficulties as a potential mechanism linking ADHD and depressive symptoms (58). In our serial mediation results, depressive symptoms were not explained by difficulties in inhibitory control, but they significantly predict aggressive behaviours, suggesting that ADHD children may tend to cope with persistent negative thoughts and emotions in a maladaptive way, through manifestations of impulsive aggression (65).

Moreover, our study provides further support for the conceptualisation of emotion dysregulation as a transdiagnostic factor of psychopathology, being a predictor of both depressive symptoms and aggressive behaviours (43, 49, 50). Likewise, it is also worth mentioning the dual pathway model developed by Sonuga-Barke (78), which emphasises the role of motivational and emotional aspects in understanding the clinical phenotype of ADHD, along with the executive dysfunction model (24). This model points to a distinction between “cool” and “hot” executive functions, in which “cool” executive functions are characterised by “purely” cognitive functions, while “hot” are related to the emotional and motivational aspects of executive functioning, implied when a situation is emotionally relevant (79). Concordant with other studies that signal the important contribution of “hot” executive functions for the characterisation of ADHD (80), our study sends out the message that emotional aspects of the disorder go beyond the cognitive dimension, at least with regard to aggressive behaviours.

Some limitations of this study should be highlighted. All the variables analysed in our study were measured considering the report of a single informant (usually the mother) representing the child’s behaviour in only one of their principal developmental microsystems. Also, we did not assess other possible variables, such as temperament and parenting practices, that may have a role in explaining the relationship between inhibitory control difficulties and aggressive behaviours. Our sample was recruited by convenience, which may bias the results as it represents parents and children who are motivated and who volunteer to participate in research. Therefore, we caution against generalising these results to different samples. The small sample size also prevents the generalisation of our results. More importantly, this is a cross-sectional study, with all variables assessed at one time point, so no causal pathways can be established. Further studies should address these issues by adopting a longitudinal design with a larger sample size and considering other informants and selection methods.

The outcomes of this study have some significant strengths and practical implications. Firstly, inhibitory control difficulties, emotion dysregulation, depressive symptoms, and aggressive behaviour were measured dimensionally, allowing all individuals to be considered along a continuum of symptoms. Also, inhibitory control difficulties, in particular, were measured ecologically, which has been suggested to be a more reliable way to capture the challenges in this cognitive dimension compared to, for example, performance tasks (81–83). Furthermore, most of the variables were assessed with different questionnaires, which prevented the common overlapping between subscales of a single questionnaire. As noted above, our findings suggest that emotion dysregulation has a crucial role in explaining the relationship between deficits in inhibitory control and aggressive behaviour, which convey important messages for professionals working directly with these children (e.g., paediatricians, psychologists, teachers, etc.). This result may inform future clinical and school intervention plans because it suggests that aggressive behaviour in ADHD is largely a result of the inability of the child to appropriately regulate their emotions, rather than a behaviour that entirely depends on their will. In fact, it is a transdiagnostic process, which is biologically rooted, that underlies the aggressive behaviour that is so often socially blamed. Though biologically rooted, this process can be supported and modelled, especially in childhood, by adults who function as co-regulatory agents, allowing the child to develop their emotion regulation skills through behaviour modelling by the adult.

## Conclusion

Despite the extensive research on children with ADHD, our study contributes to the field by investigating a novel aspect: the potential mediating role of emotion dysregulation and depressive symptoms in the link between inhibitory control deficits and impulsive aggressive behaviour. By integrating theoretical frameworks and empirical evidence, our research advances understanding of the complex interplay among these variables, providing a nuanced perspective on the mechanisms underlying aggressive behaviour in ADHD. These novel insights have implications for future intervention plans, suggesting the importance of tailored approaches aimed at fostering emotion regulation skills to mitigate and/or manage aggression in children with ADHD.

## Data availability statement

The raw data supporting the conclusions of this article will be made available by the authors, without undue reservation.

## Ethics statement

The studies involving humans were approved by the Ethics Committee of the University Hospital Centre of São João (CHUSJ). The studies were conducted in accordance with the local legislation and institutional requirements. Written informed consent for participation in this study was provided by the participants’ legal guardians/next of kin.

## Author contributions

SM: Writing – original draft, Methodology, Funding acquisition, Conceptualization. TC: Writing – original draft, Visualization, Methodology, Investigation, Formal analysis. MG: Writing – review & editing, Supervision, Resources. BS-M: Writing – review & editing, Supervision, Project administration. JF-G: Writing – review & editing, Supervision, Project administration, Methodology, Conceptualization.
